# Global shortage of technical agars: back to basics (resource management)

**DOI:** 10.1007/s10811-018-1425-2

**Published:** 2018-05-01

**Authors:** Rui Santos, Ricardo A. Melo

**Affiliations:** 1 Center of Marine Sciences, CCMAR, Algae-Marine Plant Ecology, University of Algarve, Faro, Portugal; 2 MARE—Marine and Environmental Sciences Centre, Faculdade de Ciências, Universidade de Lisboa, 1749-016 Lisbon, Portugal

**Keywords:** *Gelidium*, *Pterocladiella*, Harvest statistics, Resource management

## Abstract

Bacteriological and technical agars are in short supply with potential consequences for research, public health, and clinical labs around the world. To diagnose bottlenecks and sustainability problems that may be putting the industry at risk, we analyzed the available time series for the global landings of *Gelidium*, the most important raw materials for the industry. Data on the harvest of *Gelidium* spp. have been reported since1912, when Japan was the only producer. After World War II the diversification of harvested species and producing countries resulted in a strong increase in global landings. Maximum harvest yields of almost 60,000 t year^−1^ in the 1960s were sustained until the 1980s, after which landings decreased continuously to the present. In the 2010s, a reduction in the global production to about 25,000 t year^−1^ was observed, which was lower than the yields of the 1950s. Landings by important producers such as Japan, Korea, Spain, and Portugal have collapsed. This is the ultimate cause of the present shortage of bacteriological and technical agars. However, an important factor at play is the concentration of the global landings of *Gelidium* in Morocco, as its relative contribution increased from 23% in the 1960s to the present 82%. Two specific bottlenecks were identified: restrictive export quotas of unprocessed *Gelidium* in favor of the national agar industry and resource management regulations that were apparently not enforced resulting in over-harvesting and resource decline. The global industry may well be dependent on resource management basics. Simple harvest statistics must be gathered such as the harvest effort and the variation of harvest yields along the harvest season. We discuss how this information is fundamental to manage the resource. The available harvest statistics are generally poor and limited and vary significantly among different sources of data. Probable confusions between dry and wet weight reporting and poor discrimination of the species harvested need to be resolved.

## Introduction

Natural populations of species of the red algal genus *Gelidium* (Rhodophyta) are exploited worldwide for the extraction of technical agars (e.g., bacteriological agar and agarose) and constitute the most important source of raw material for the industry as *Gelidium* aquaculture has not been feasible at large scale (Melo et al. 1991). Even though the agar extracted from *Gelidium* presently represents only about 1.6% of the world phycocolloid production (Porse and Rudolph 2017), its natural high gelling strength and low gelling temperatures make it difficult to be replaced by agars extracted from other species. Higher grade, purified agars used in pharmacological, biomedical, biotechnological, and some other specific applications are extracted exclusively from *Gelidium* (Armisén 1991). The demand for bacteriological agar and agarose from *Gelidium* has increased from 250 and 15 t, respectively (Santos 1993), to about 700 and 50 t. The demand is presently higher than the offer as recently highlighted by Callaway (2015). Bacteriological and technical agars are presently in short supply, pushing wholesale prices to an all-time high of around US$35–45 per kilogram, nearly triple the price before the scarcities began.

In face of this situation we present here a global assessment of the world exploitation of *Gelidium* natural resources in order to diagnose sustainability bottlenecks that may be putting at risk the *Gelidium* agar industry. Unfortunately, not much published information is available on the subject. Within a more general assessment of worldwide distribution of commercial seaweeds, McHugh (1991) presented a point in time estimation of the world harvest of *Gelidium* based on government statistics and/or information received from industrial, academic, and government organizations. At that time about 50% of the world’s *Gelidium* landings originated from Spain, Portugal, and Morocco; Japan and South Korea contributed about 14% each, Mexico 10%, and substantial quantities were also collected in Indonesia (about 7%). Later, Melo (1998) presented a detailed analysis of the exploitation of *Gelidium* spp. in Spain, Portugal, and Morocco and concluded that during the 1990s, landings decreased in Portugal and increased in Spain and Morocco. A small series of data from 1990 to 1996 was also presented for the harvest of *Gelidium pristoides* in South Africa. Melo (1998) also reported that in Japan, the *Gelidium* harvest had ceased, largely due to the low prices of imported agarophytes; in Mexico, there was a 66% decrease of landings and no further data were presented for South Korea.

Given the unclear global picture for the exploitation of *Gelidium* spp., the objectives of this article were to make a thorough compilation of the available, country by country, time series data for *Gelidium* spp. landings. A global analysis of the state of the resource was carried out and bottlenecks for sustainability were clearly revealed. The few available data on commercial agar production were used in order to estimate the industrial yield of the hydrocolloid and to calculate the world agar production from *Gelidium*. For detailed descriptions of harvest methods and cultivation attempts of *Gelidium* species around the world, see previous reports by Santelices (1988), Santos and Duarte (1991), Melo (1998), and McHugh (2003). More recently, Porse and Rudolph (2017) assessed the present situation of the seaweed hydrocolloid industry, comprising agar, alginate, and carrageenans.

## Methods

The compilation of *Gelidium* spp. harvest yields and agar production/export data is a difficult task as most of the information is scattered and not readily available, or is with-held due to commercial concerns. As often happens, the absolute values reported by different sources do not match. To reconstruct the time series of *Gelidium* landings, we used an adaptation of the catch reconstruction approach used by Pauly (1998) and Pauly and Zeller (2016) for fishery data. This required comparisons of data from various sources, interpolations, and bold assumptions justified by the unacceptability of the alternatives, for example, data known to be incompatible with empirical data and historic records. In some cases, we contrasted the data obtained directly from country-specific sources with the data provided by FishStat database, published by the Fisheries and Aquaculture Department, Food and Agriculture Organization of the United Nations, which provides statistical time series of world fisheries, including seaweeds (aquatic plants) (FAO 2016).

All data presented here are in wet weight. When necessary, a conversion factor of 3-fold was used to estimate wet weight from dry weight values, following the conversion used by FAO (2016). The annual industrial agar yields of *Gelidium*, i.e., the ratio between agar production and dry weight landings, were estimated whenever yearly agar production data were available.

## Results

### Japan

**Fig. 1:**
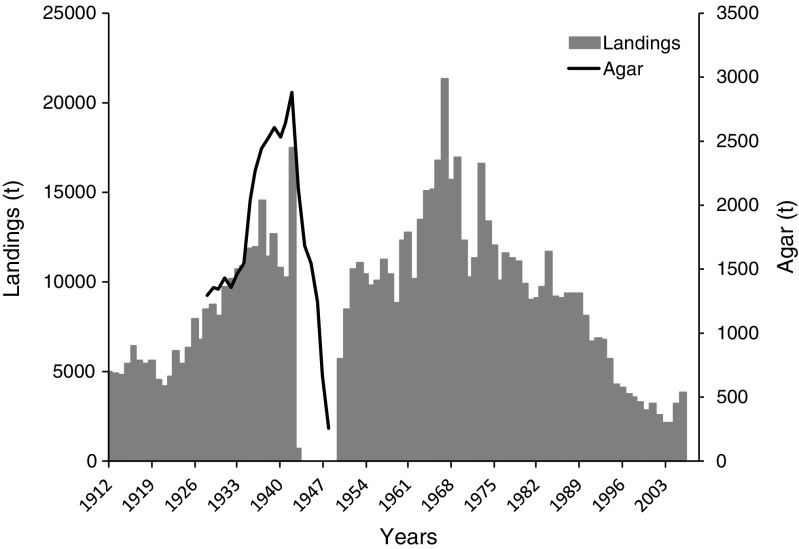
*Gelidium* spp. landings and agar production in Japan

Japan is the country of origin for the original use of *Gelidium* spp. for the extraction of agar. The most important species harvested has been *Gelidium amansii* even though other species were harvested such as *G. elegans* (Fujita et al. 2006). The seaweeds were harvested by hand in the intertidal or by rakes in shallow subtidal areas (Fujita et al. 2006). Figure 1 presents a reconstructed time series of landings, using data kindly provided by Daisuke Fujita and data from FAO (2016). The first available data on landings go back to 1912. Landings increased consistently to annual values up to about 15,000 t at the early 1940s. By World War II, Japanese *Gelidium* was the main world source of raw material for agar. No data were available for the late 1940s, when landings probably crashed as evidenced from agar production. During the 1950s to the 1970s, landings were maintained at high levels. The relatively constant annual landings throughout five decades (except for the 1940s) are also consistent with the data provided by Suto (1974), which averaged 16,200 t. From the 1970s, to the present, landings steadily declined to minimum values of 2–3000 t. The last available data for the harvest of red seaweeds in Japan (FAO 2016) were for 2005 and 2006 (Fig. 1).

The available time series data on agar production show that it followed the increasing trend of landings from 1910s to the 1930s, followed by a sharp decrease in the 1940s. The average industrial agar yield, taking in consideration all of the available data, was 19%.

### Republic of Korea

*Gelidium amansii* has been the main species harvested in Republic of Korea, but smaller quantities of other *Gelidium* species were also harvested (Lee et al. 2011). The time series of landings is highly variable, probably due to poor reporting whether the harvest data were in dry or wet weight. In fact, available dry weight data of the 1990s and 2000s from both Lee et al. (2011) and the Ministry of Maritime Affairs and Fisheries (MOMAF) are consistent with each other and very similar to those of FAO (2016), which supposedly reports data as wet weight. McHugh (1991) reported that landings were about 2900 dry t, which was consistent with about 8000 wet t, as reported by FAO (2016).

**Fig. 2:**
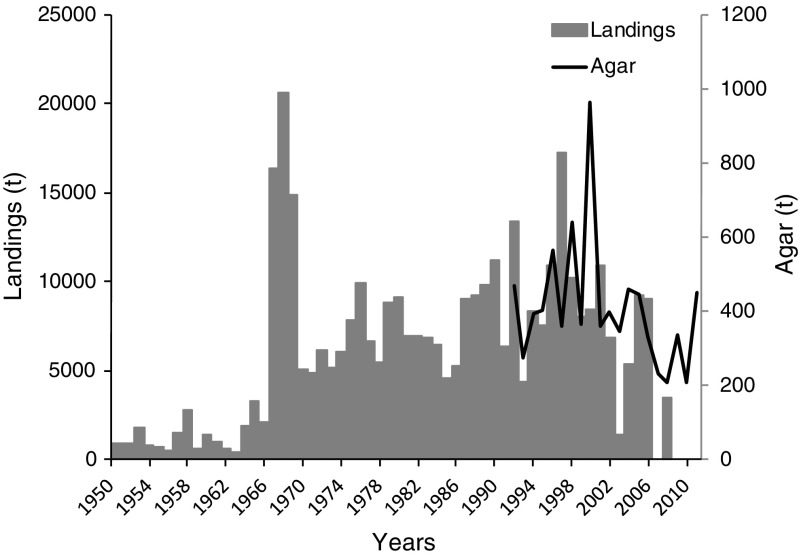
*Gelidium* spp. landings and agar production in the Republic of Korea

Figure 2 includes the wet weight data reported by FAO (2016) from 1950 to 1995 and from then on, the reconstructed data considered three sources of information, namely Lee et al. (2011), FAO (2016), and MOMAF. An overall trend of increasing landings was observed from 1950 to 1990s followed by a decreasing trend to the present.

Available data on the production of agar from the Korean Statistical Information Service (KOSIS) showed high year to year variations and a low relationship with landings (Fig. 2). The average industrial agar yield, taking in consideration all available data, was 11%.

### Spain

There are four harvest zones for *Gelidium corneum* in Spain. These are located in the North Atlantic coast, which correspond to the boundaries of the autonomous regions of Galicia, Asturias, Cantabria, and Euskadi (Basque Country). The traditional harvest method has been to gather storm tossed algae along the beaches (Juanes and Borja 1991). This method contributed about 90% of the total landings. Harvest by divers operating from small boats has also been used, mainly in Asturias where *G. corneum* has the common name of “ocle.” In Euskadi, scuba divers operated only in the 1970s and 1990s (Borja et al. 2013). In the early 1990s, 10 boats operated in Asturias, 3 in Cantabria, and 2 in Euskadi. In the 2000s, 18 boats were operating in Asturias and 10 in Cantabria. The harvest of storm tossed *G. corneum* was made mainly in Asturias, Cantabria, and Euskadi.

**Fig. 3:**
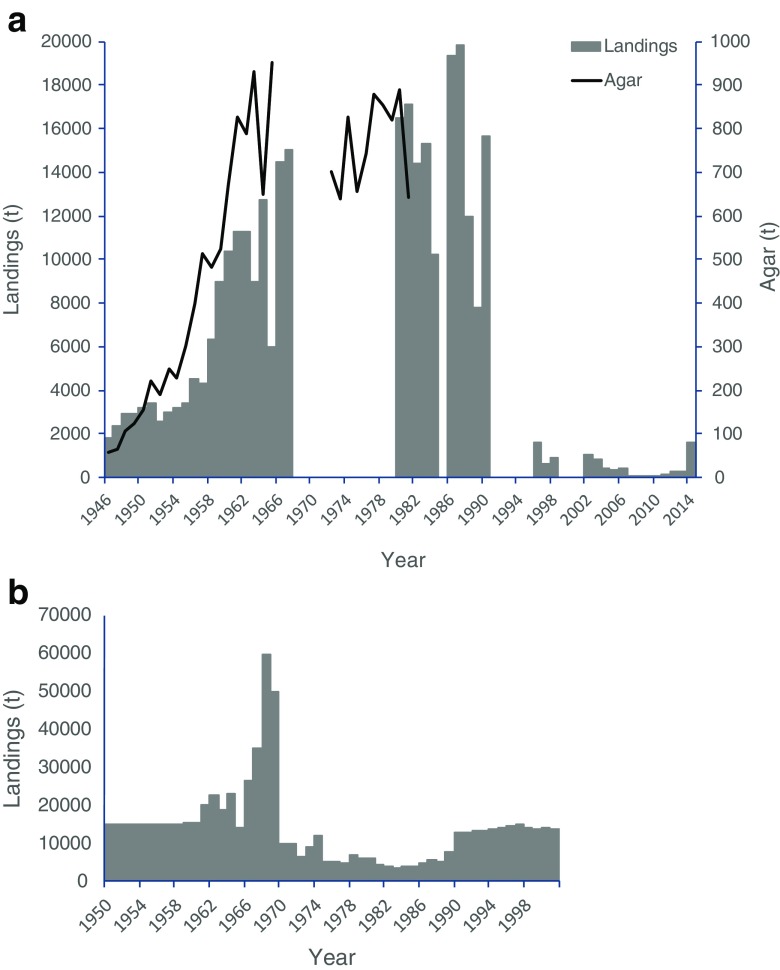
*Gelidium* spp. landings and agar production in Spain. **a** Reconstructed data. **b** Data from FAO (2016)

The time series presented in Fig. 3 was reconstructed using data kindly provided by Rafael Armisén, from Hispanagar SA, and from various other sources, including research colleagues and online information from the autonomous Spanish regions. As in Portugal, the harvest of *G. corneum* began during World War II and was forced by a shortage of imports from Japan, which until then had been the major source of agar for Europe and North America. Landings increased consistently until the 1980s, when the maximum resource exploitation was maintained at about 6000 t. During the 1990s the harvest crashed to very low levels of around 300 t and did not recover during the 2000s. At present harvest is done in Asturias, Galicia, and Cantabria.

The available time series of FAO (2016), presented in Fig. 3b, is quite different from the reconstructed one. Initially, stable values of 15,000 t during the 1950s increased to a peak in the late 1960s at values about three times the maximum reconstructed values, again indicating potential confusion between dry and wet weights. Landings during the 1990s were one order of magnitude higher than the reconstructed ones. Landings reported by FAO (2016) in the 2010s were included in Fig. 3a, as they were in accordance to those reported by other sources.

Agar production appears to have followed the seaweed harvest pattern, maintaining high production values of about 800 t year^−1^, during the 1960s to the1980s. The average industrial agar yield was 20%. According to Iberagar SA, a Portuguese hydrocolloid company in the Hispanagar SA group, the agar yield of the Spanish industry is around 15–17%.

### Portugal

**Fig. 4:**
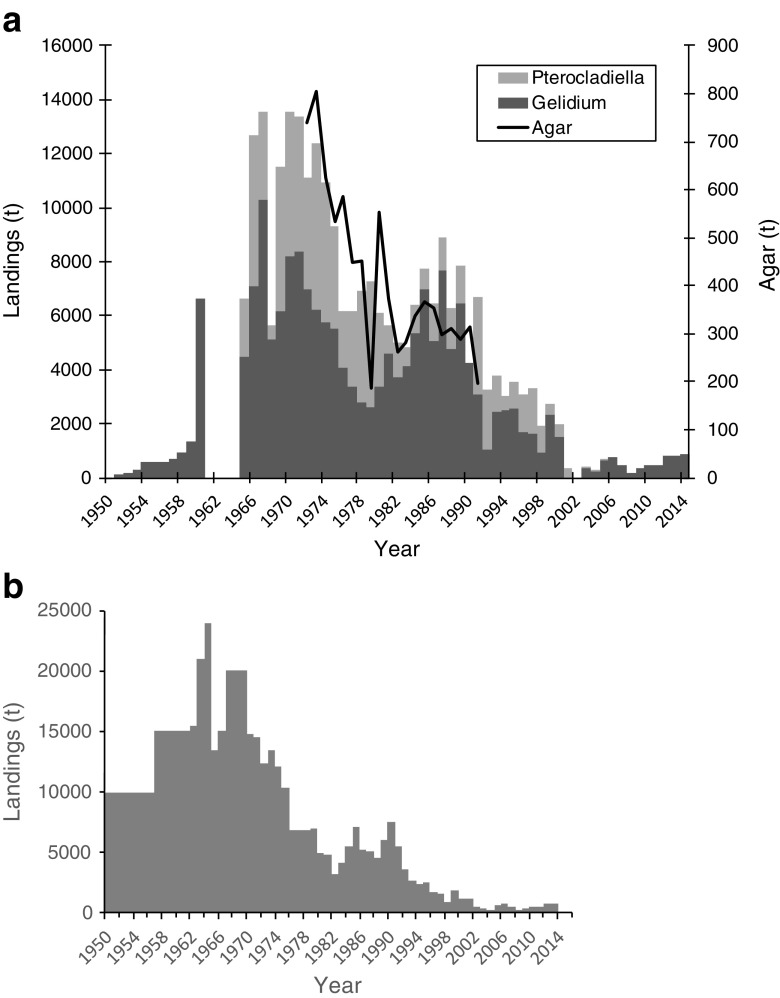
*Gelidium* spp. and *Pterocladiella* landings and agar production in Portugal. **a** Reconstructed data. **b** Data from FAO (2016)

*Gelidium corneum* has been harvested in Portugal since World War II. The harvest by divers operating from small boats did not start until the early 1960s (Santos and Duarte 1991). In the 1960s, another agarophyte, *Pterocladiella capillacea*, began to be harvested in the Azores Islands. The national landings increased consistently until the early 1970s (Fig. 4); it then decreased in the late 1970s and early 1980s (mostly in the mainland) probably due to socio-economic constraints related to the political change from dictatorship to democracy in 1974 and increased again to a second period of high landings during the late 1980s and early 1990s. After that, the exploitation of agarophytes decreased continuously to very low levels. At present, the harvest is only carried out in harvest zone 3, one of the six harvest zones that the Portuguese coast is divided into (see harvest zones in Santos and Duarte 1991). The general trend of FAO (2016) data on red seaweeds (Fig. 4b), subtracted from the Portuguese carrageenophyte landings (data not shown), is similar to the previous data, except that the values from the 1950s and 1960s are highly over-estimated.

The production of agar in Portugal followed the general trend of the raw material landings (Fig. 4a). The global average agar yield of the industry was 17%.

### Morocco

**Fig. 5:**
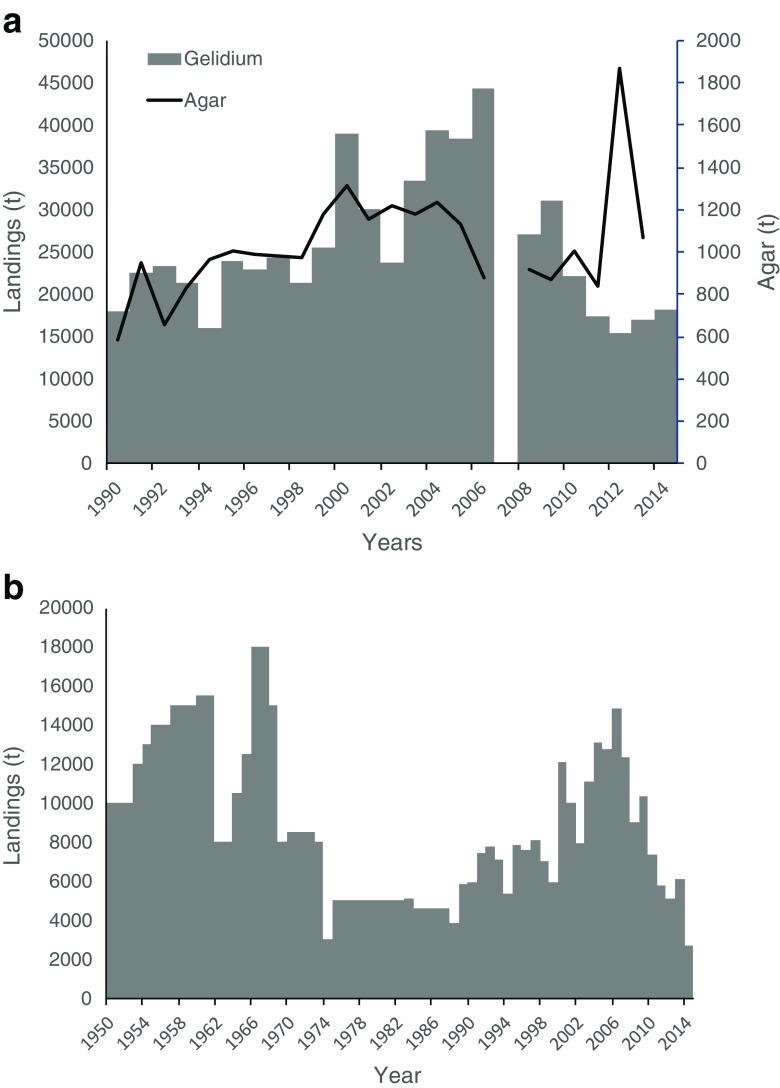
*Gelidium* spp. landings in Morocco. **a** Reconstructed data. **b** Data from FAO (2016)

*Gelidium corneum* has been harvested in Morocco since the 1950s in the intertidal, mostly by women, children, and older people who gather the storm-tossed plants, in the shallow subtidal by man snorkeling with buoys and in the subtidal by hookah divers operating from boats (Givernaud and Mouradi 2006). The annual landings reported by the Ministère de la Agriculture et de la Pêche Maritime showed relatively stable landings during the 1990s, an increase to a maximum of 44,000 t in 2006 and a decrease to half of that in the present (Fig. 5a). The same trend was revealed by FAO (2016) data (Fig. 5b) during this time period. However, the magnitude of values reported is exactly 3-fold lower, strongly suggesting that dry weight data were reported instead of wet weight.

There were no available data on agar production, from the harvested biomass, but the agar export statistics can be used as a proxy of the agar production since most of the agar produced by the country was exported. Agar exports generally followed the trend of seaweed landings, except in recent years when landings were low and agar exports were high (Fig. 5a). Considering that all the seaweed that was not exported (data not shown) was processed by the national agar industry and that all the agar that was produced was exported; the average industrial yield of *G. corneum* was 17%.

### Mexico

**Fig. 6:**
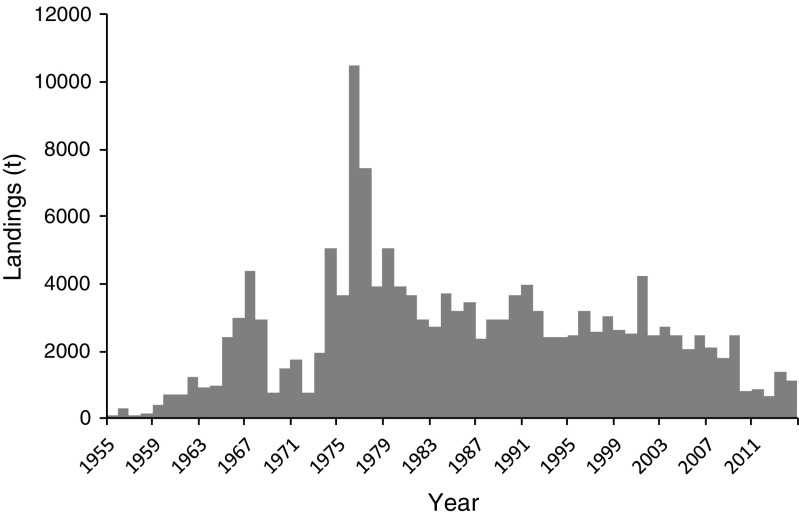
*Gelidium* spp. landings in Mexico

*Gelidium robustum* (common name “sargazo rojo”) has been harvested along the central coast of Baja California, Mexico, by hooka divers operating from small boats since 1955 (Fig. 6). The reconstructed data are mostly based on INAPESCA (2012) and open access online fisheries statistics (CONAPESCA-SAGARPA 2015). This is because FAO (2016) data included other red seaweeds, both agarophytes and carrageenophytes, which in the case of Mexico were an important fraction of the harvest. An expected initial phase of increasing landings corresponding to the development of the fishery was observed from 1955 to a peak in 1967 at 4500 t. This was followed by a decline during the early 1970s with a high peak at 9200 t in the late 1970s. Overall, the landings were maintained, after the initial development phase, to the 2000s, at a level around 3300 t. The 2010s saw the annual landings decrease to about 1000 t.

The few available data on agar exports, from 1990 to 1992, indicated that the industrial yield of agar from *G. robustum* was about 16%, which was similar to *G. corneum* agar yields in the Northeast Atlantic (17–20%). A personal communication from Iberagar SA puts the Mexican agar yield at around 10–12%.

### Chile

**Fig. 7:**
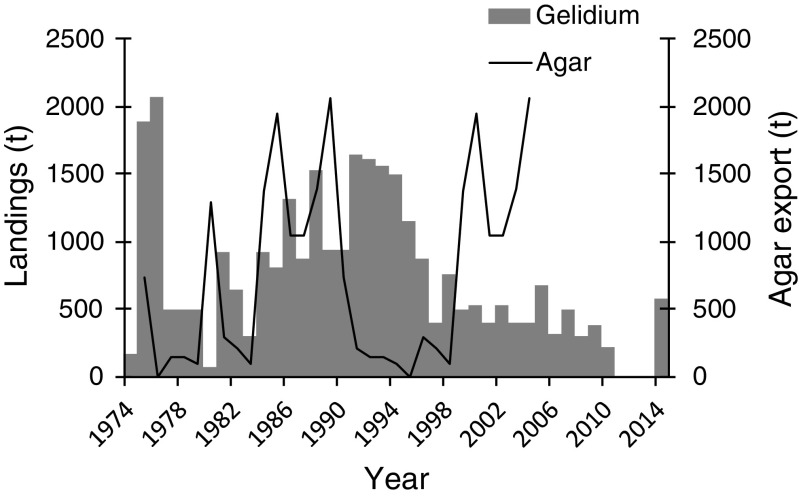
*Gelidium* spp. landings in Chile

Three species of *Gelidium* (commonly named as “chasca”), i.e., *G. lingulatum*, *G. chilense*, and *G. rex*, are harvested by hand in the intertidal zone of Chile. Published reports indicated that the landings in the 1980s and early 1990s were around 400–500 t (McHugh 1991; Avila and Seguel 1993). The reconstructed data (Fig. 7) were gathered from FAO (2016), IFOP (2016), and SERNAPESCA (2017) online databases. Increased exploitation of the resources to maximum values was apparent in the 1990s, except during the 1974–1975 period when landings were exceptionally high. Landings decreased steadily from the 1990s to the present, at about 80–100 t year^−1^ (Iberagar SA, pers. comm.).

The quantities of exported agar were actually higher than the reported *Gelidium* landings and increased throughout the 2000s, at a time when the reported landings of *Gelidium* were declining. This was a clear indication that most of the agar produced in Chile was not derived from native *Gelidium* species, but rather from *Gracilaria* species.

### South Africa

**Fig. 8:**
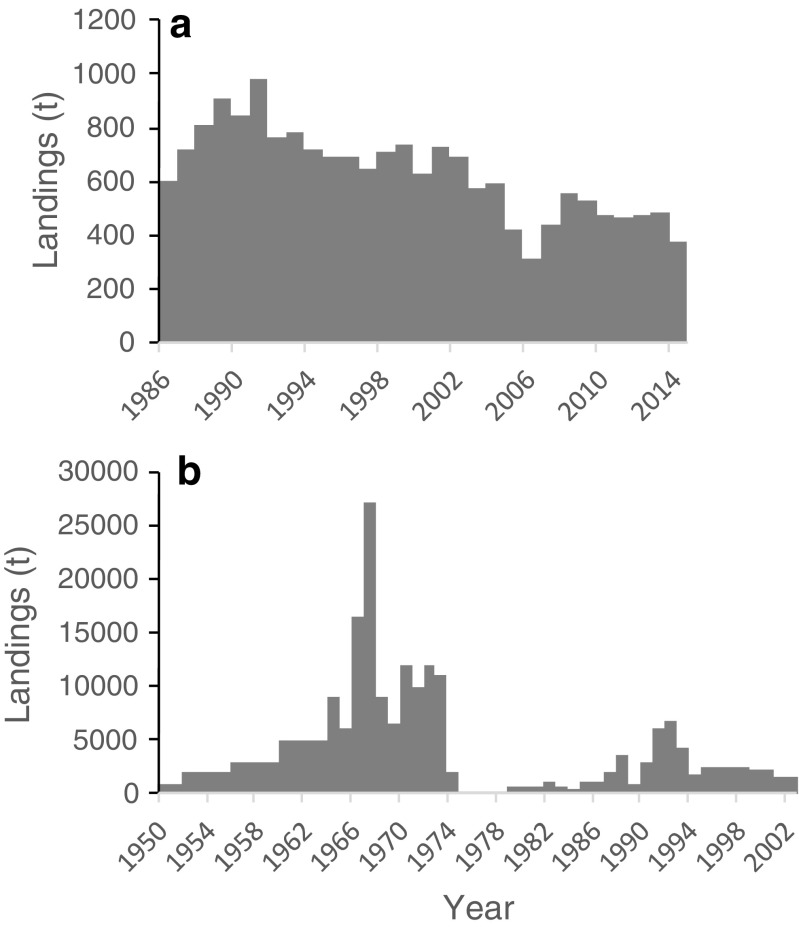
*Gelidium* spp. landings in South Africa. **a** Reconstructed data. **b** Data from FAO (2016)

Commercial harvest of *Gelidium* species (*G. pristoides*, *G. abbottiorum G. pteridifolium*, and *G. capense*) has been undertaken in South Africa since at least 1957, and practically, all of it was exported to Japan and Korea (Anderson et al. 2003). The landings since the late 1980s showed a slow decline from about 800 t to the present 500 t (Fig. 8). FAO (2016) reported landings of red seaweeds from 1950 to 2002, but they are very high, including seaweed species other than *Gelidium* spp. The contribution of *Gelidium* landings between 1986 and 2002 (Robert J. Anderson pers. comm.) to the total landings reported by FAO (2016) was, on average, 34%.

### Taiwan

**Fig. 9:**
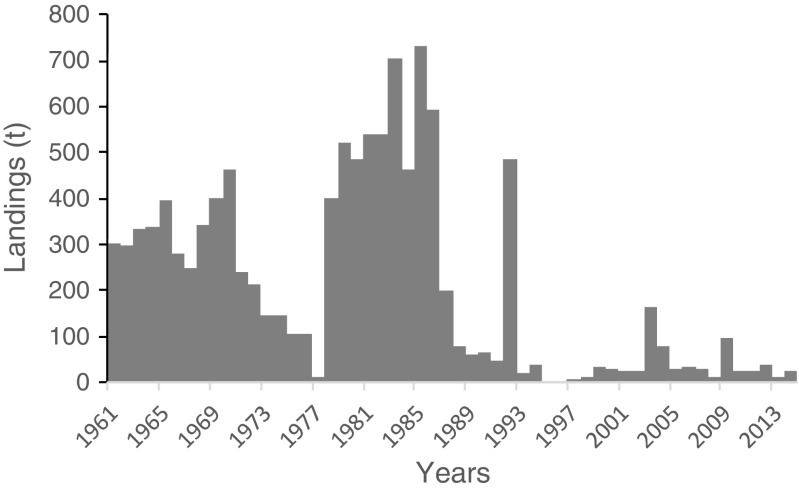
*Gelidium* spp. landings in Taiwan

Taiwanese *Gelidium* landings have been reported by FAO (2016) since 1961 (Fig. 9). Landings were sustained in the 1960s at around 300 t, and decreased to 1977, recovered to the highest levels of 700 t in 1985 and again declined to the present residual levels of about 25 t. The main species harvested was *G. amansii*, growing on the north and northeast coasts. The species has also been cultivated in ponds, but no longer at present (Iberagar SA, pers. comm.).

### Other countries

*Gelidium corneum* has been harvested along the French, Basque coast, mostly along a 20 km stretch of coast at Hendaye, near the border with Spain. Drifting masses of this seaweed were traditionally harvested in the autumn at sea by trawlers and along the shore by hand. Harvested quantities of 400–1200 t were reported by Kaas (1998), Dejeans (2011) reported 736 t in 2010 and 1116 t in 2011, and Dejeans (2016) reported 1800 t in 2015. These yields seem to be over-estimates in the face of the reduced extent of the harvest zone and probably include sand and other seaweeds. FAO (2016) data of red seaweeds landings in France showed a yearly average of 4000 t from 1953 to 1995, decreasing to 1000 t in the 2000s. These data certainly include carrageenophyte species.

Commercial harvesting of *Gelidium madagascariense* [included in the new genus *Orthogonacladia*, as *O. madagascariense* by Boo et al. (2016)] was reported for the southeast coast of Madagascar, in an area south of Fort Dauphin, since the early 1980s Mollion (2006). It was mostly based on collection of drift material on the shore, although a small harvest (~ 10%) was carried out by shore divers at small depths. The reported harvests, which were all exported to Japan, peaked at 900 t in 2000, and then declined and stopped.

*Gelidium serrulatum* has been harvested in Trinidad and Tobago’s northern coastline by local communities and used in many households. The harvest, carried out mainly in August, was described as becoming “scarcer,” which prompted the development of a local management project supported by the Caribbean Environmental Resources Institute (http://www.canari.org/), the UK Department for International Development, and the United Nations Development Programme.

*Pterocladia lucida*, mostly, and *Pterocladiella capillacea* (5%) have been harvested by hand in New Zealand since 1943, mainly along the coasts of Wairarapa, Hawke Bay, and Poverty Bay (Booth and Cox 2003). In 1944, the harvest was 150 t; during the 1970s, the average harvest was 109 t year^−1^ (Luxton and Courtney 1987). In the 1980s, the harvest was taken mainly from drift or beach-cast plants (69–75%) whereas 25–31% was plucked in the intertidal or by diving (Luxton and Courtney 1987). Schiel and Nelson (1990) reported a level of 250 t year^−1^, and landings have been stable around that value since then (FAO 2016). A New Zealand business group has been investing in modern processing of *Pterocladiella* since 2011, when the only local factory, operating since 1978, was purchased (http://www.nzmanukagroup.com/group/nz-seaweeds/). A new agar seaweed processing facility was opened in early 2017.

Prospecting for agarophytes in India started during World War II and produced a cottage industry based mainly on *Gelidiella acerosa* that yielded bacteriological/ pharmaceutical grade agars, whereas other agarophytes were used for food grade agars (Baby Ushakiran et al. 2014). The annual harvests of *G. acerosa* in the Gulf of Mannar in 2003–2012 yielded 1100–1500 t resulting in 50–90 t of agar (Ganesan et al. 2014). *G. acerosa* is also abundant in areas of the Philippines (Rollon et al. 2003) and Thailand (Fujimoto et al. 2014), but we were not able to gather any information on a putative harvest. As well, relevant amounts of *G. latifolium* have been harvested in Indonesia (Soegiarto 1990), picked by hand either at low tide or by snorkeling in shallow waters, but we did not find any time series data on annual landings. Mc. Hugh (1991) reported 1400 t of dry weight harvested over a wide area, including the southern coast of Java, the islands between Java and Timor, Sumatra, and several areas to the north and east of Timor. Porse and Rudolph (2017) reported the harvest of 4000 t and 3500 t (dry weight) of *Gelidium* in 2009 and 2015, respectively, in the set of countries of Japan, Korea, and Indonesia.

### Global assessment

**Fig. 10:**
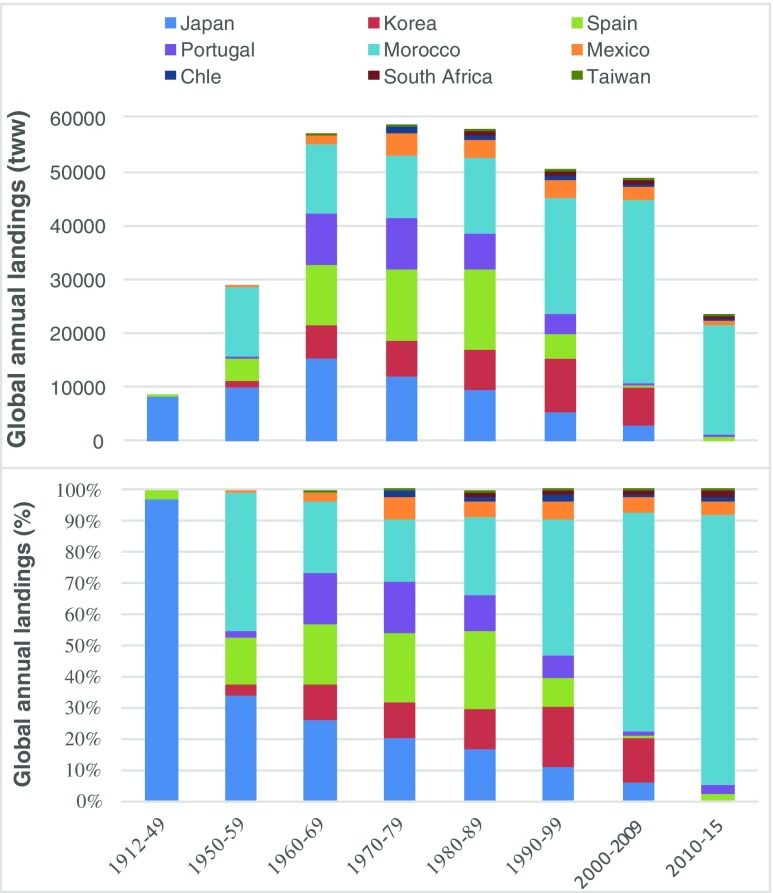
World decadal landings of *Gelidium* spp.

Data on the exploitation of the natural resources of *Gelidium* spp. on a worldwide basis are available back to 1912, when Japan was the only producer (Fig. 10). This situation was maintained until World War II when other countries started the exploration of their indigenous *Gelidium* and alternative agarophyte resources in reaction to the shortages of agar supplied by Japan due to interrupted trade (Tseng 1944). As a result, global landings increased 3-fold during the 1950s. The diversification of producing countries, particularly Korea, Spain, Portugal, and Morocco, pushed the global landings to the maximum values of almost 60,000 t year^−1^ during the 1960s. The global development of the fishery coincided with the decrease of the Japan’s role as a major producer, from the initial 100% to 34% in the 1950s and 26% in the 1960s. Maximum global landings of *Gelidium* spp. were sustained for three decades until the 1990s when landings decreased continuously to the present. Important changes in the relative contribution of producers happened during this period, when landings decreased in most countries and the global production became concentrated in Morocco. The relative contribution of this country increased from 23% in the 1960s to the present 82%. During the 2010s, there was a sharp reduction in the global production to about 25,000 t year^−1^, a value lower than the 1950s. The landings of important producers such as Japan, Korea, Spain, and Portugal have collapsed. Landings in Taiwan, Chile, and South Africa declined as well, and only Mexico maintained historical levels of harvest.

The global production of agar, considering an average industrial agar yield of 17%, peaked in the 1980s at almost 10,000 t. In the 2010s, only 4000 t of agar was extracted from *Gelidium*.

## Discussion

The analysis of the global trends of *Gelidium* spp. landings showed that the production of raw material for the agar industry clearly shifted from a multi-species/multi-country exploitation within the period of maximum landings (1960–1980s) when about 44% of the world’s raw material originated from *Gelidium* species other than *G. corneum* to the present single species/country exploitation where *G. corneum* from Morocco represents 82% of the world’s production. There is no indication that the main factor influencing this shift has been the over-exploitation of natural resources but rather socio-economic reasons. In countries such as Japan, Spain, and Portugal which represented 56% of the world’s production in the 1960s–1980s, evidence has been reported that sustains this statement. In Japan, Fujita et al. (2006) reported that the major reason for the reduction was the increase of imports of cheaper raw material from other countries such as Morocco that led to a loss of interest of the traditional harvesters. Melo (1998) reported that while in 1990/1991, there were no exports of *G. corneum* from Morocco to Asia; since 1992, they became increasingly important, representing about 60% of the country’s total production in 1996. The Asian exports of Moroccan *G. corneum* during the early 2000s were still about 53%.

In Spain, where the harvest has been based on the gathering of cast seaweed resulting from natural breakage during autumn storms, the observed crash in landings since early 1990s could not have been caused by over-exploitation of resources. The poor quality of storm-tossed seaweeds, highly contaminated with sand and other seaweeds, and the large fluctuations in yearly landings posed a serious problem to the industry that required a steady input of high-quality raw material. Quintano et al. (2017) suggested that light and nutrient availability and wave energy may be involved in the reduction of *G. corneum* beds in Euskadi. These factors explain why the industry started importing the better quality (and lower priced) Moroccan *Gelidium*, which were hand-picked by divers. Melo (1998) reported that in the early 1990s, more than 80% of the Moroccan exports of *G. corneum* were to Spain. Following the Prestige oil-spill in 2002, the *Gelidium* harvest was closed and Spanish agar factories increased the import of raw material from Morocco. As a consequence the price of locally harvested *G. corneum* dropped sharply and many harvesters abandoned the activity (A. Borja, pers. comm.). In the 2010s the whole sector declined in Spain. There are no statistics gathered at a national or regional level, both for landings and agar production, and the industry is losing its former world importance, particularly for production of bacteriological-grade agar (J. Salinas, pers. comm.). Only the production of agarose is still important.

As in Japan and Spain, the *Gelidium* landings in Portugal crashed during the 1990s mainly due to a drop in internal demand, resulting in the closure of two of the three Portuguese agar processing units (Melo 1998). Increased difficulties in selling the harvested seaweed led to the loss of interest of the harvesters. In addition, there is evidence of a decrease in the standing stocks of two of the three main harvest areas due to over-exploitation (Santos et al. 2003). The activity did not recover since then, even though the steep price rise of technical agars in 2015–2016 (Iberagar SA, pers. comm.) fostered the harvest effort in the remaining harvest zone (zone 3; see Santos and Duarte 1991).

The harvest decline in Japan, Spain, and Portugal was balanced by the increased production in Morocco during the 1990s and 2000s. However, in the 2010s, there was again a decline in Moroccan landings and global production crashed to those of the initial phase of exploitation in the 1950s (about 25,000 t). This was 42% less than the maximum sustained landings of the 1960–1990s period. The present historical lowest *Gelidium* spp. global harvest is the ultimate cause for the shortage of bacteriological and technical agars that are pushing wholesale prices to the triple.

The dependence of the global industry on one country’s production, Morocco, is a serious threat as natural resources have not been well managed. An effort of Moroccan scientists was made to evaluate the standing stocks of *Gelidium* (Givernaud et al. 2005), to assess its reproductive biology (Zidane et al. 2006), and to investigate the possibility of re-stocking over-harvested beds (Givernaud et al. 2003). However, these efforts were doomed to failure as resource management regulations were not effectively enforced. The number of unlicensed harvesters was out of control and the harvest season was not being respected (Givernaud and Mouradi 2006). Even though landings increased in the 1990s and 2000s and new *G. corneum* beds were exploited in southern Morocco, the number of diving harvesters, most of them unlicensed, increased 10-fold. Only 10–20% of the harvest was made by boats owned by the agar companies, under official management regulations. Evidence of over-exploitation of the Moroccan beds of *Gelidium* was presented by Givernaud et al. (2005). This is an unfortunate confirmation of the necessity of enforcing resource management regulations and particularly to improve the gathering and analysis of harvest statistics.

The worldwide data on *Gelidium* spp. landings are generally available, but their quality must be improved. As we have revealed here, significant differences are observed when different sources of data are analyzed. Probable confusions between dry and wet weight reporting and poor discrimination of the species harvested need to be resolved. On the other hand, the simple reporting of harvested weight is not enough to assess over-exploitation. It is crucial to quantify the harvest effort of each bed to evaluate the catch per unit of effort (CPUE), an essential statistic for *Gelidium* resource management as shown by Santos et al. (2003). Harvest parameters such as the species catchability and the pre- and post-harvest standing stocks can be easily derived from the relationship between the catch per unit of effort and the cumulative landings throughout the harvest season (Santos et al. 2003). Based on these parameters, bed-specific exploitation rates may be defined to prevent over-exploitation and ensure a sustainable harvest. We emphasize that the gathering of bed-specific harvest data along the harvest season is much more informative and much less costly than implementing direct stock assessment sampling designs by scientific divers (Santos et al. 2003).

A good example of resource management of *Gelidium* spp. based on harvest statistics can be found in Mexico. Relationships between the CPUE and environmental factors were established (Casas-Valdez et al. 2001) allowing the forecast of standing stocks and their variation with the fluctuating environment (El Niño events). As well, estimations of maximum sustainable yields have suggested that the resource has not been over-exploited (Casas-Valdez and Lluch-Belda, 2005). Sound scientific inputs are needed to implement adequate management practices. Another good practice for the management of *Gelidium* harvest is the concession of harvest areas to companies that are responsible for managing the resource, as it has been implemented in South Africa with success, since the early 1990s (Anderson et al. 2003). The fact that our data show a consistent decreasing trend of *Gelidium* annual landings is probably not related to poor management, but to the loss of interest of operators that are now focusing on harvesting fresh kelps for feeding abalones (Troell et al. 2006).

The present shortage of bacteriological and technical agars can be an opportunity for the re-kindling of *Gelidium* harvest in those countries where the activity crashed. Also, increased interest in prospecting and evaluating new *Gelidium*-based agarophyte resources is emergent in other countries, such as *G. spinosum* in Tunisia (Ben Said et al. 2009, 2013), *G. crinale* and *Pterocladiella capillacea* in Egypt (Ibrahim et al. 2015), *G. crinale* in Argentina (Croce et al. 2015), *G. corneum* in Algeria (Nil et al. 2016), and *G. elegans* in Malaysia (Ajdari and Zarshenas 2016).

In conclusion, we emphasize the sensitivity of the global bacteriological and technical agar industries to the Moroccan harvest of *Gelidium corneum.* Two main factors are presently at play, the decline of the Moroccan resource caused by over-harvesting of commercial beds and the national enforcement of restrictive quotas for the export of the raw material. In an effort to favor the Moroccan industry of agar, up to 80% of the national landings have to be processed in-country. To assure sustainable harvesting of global *Gelidium* resources, effective management strategies need to be enforced. Efforts must be taken to implement the gathering of daily harvest yield and harvest effort data so that the changes in the catch per unit of effort throughout the harvest season can be monitored. As stated above, these simple statistics may provide estimates of both the standing stock and the exploitation rate of the resource without the need for extensive, high cost sampling assessments.
